# Discovery of *Epichloë* as novel endophytes of *Psathyrostachys lanuginosa* in China and their alkaloid profiling

**DOI:** 10.3389/fmicb.2024.1383923

**Published:** 2024-05-23

**Authors:** Taixiang Chen, Tian Wang, Mingxiang Du, Kamran Malik, Chunjie Li, Gensheng Bao

**Affiliations:** ^1^State Key Laboratory of Plateau Ecology and Agriculture, Qinghai University, Xining, China; ^2^State Key Laboratory of Herbage Improvement and Grassland Agro-ecosystems, Gansu Tech Innovation Centre of Western China Grassland Industry, College of Pastoral Agriculture Science and Technology, Lanzhou University, Lanzhou, China; ^3^Qinghai Academy of Animal and Veterinary Medicine, Xining, China

**Keywords:** endophyte, *Psathyrostachys lanuginosa*, *Epichloë bromicola*, alkaloids, vertical transmission

## Abstract

The *Epichloë* genus represents a significant group of above-ground endophytes extensively researched for their potential applications in agriculture and ecology. Additionally, *Epichloë* species synthesize bioactive alkaloids, which generally cause health problems in livestock and have detrimental effects on the performance of insect herbivores. *Psathyrostachys lanuginosa* serves as a valuable forage grass for livestock owing to its high nutritional value and resilience in adverse environmental conditions. Nevertheless, to date, no reports have documented *Epichloë* as endophytes of *P. lanuginosa*. In this study, four strains (PF5, PF9, QG2, and QG4) were isolated and identified through morphological, molecular, and phylogenetic analyses as endophytes of *P. lanuginosa*. Morphological analysis indicated colony characteristics and conidia features consistent with symbiotic *Epichloë*, with no significant differences observed in growth rates or conidia dimensions among the four strains. Phylogenetic analysis confirmed all strains as *E. bromicola*. Additionally, alkaloid biosynthetic genes were detected, revealing differences in the potential synthesis of peramine and indole diterpenoid alkaloids among strains from different geographic origins. However, all four *E. bromicola* strains exhibited similar potential for synthesizing ergot alkaloids, but not loline alkaloids. Overall, this study identified *P. lanuginosa* as a novel host for *E. bromicola* and provided insights into the alkaloid profiles of these strains, laying a solid foundation for the scientific and rational utilization of *Epichloë* resources.

## 1 Introduction

The interaction between plants and microorganisms is common in nature and plays a vital role in plant ecology and agriculture (Shalev et al., 2022). The endophytic genus *Epichloë* consists of above-ground filamentous fungal endophytes known for their host specificity. *Epichloë* primarily infects above-ground plant parts, such as seeds, stems, and sheaths, while it does not grow in the roots (Christensen et al., 2008). The grass family Poaceae is the sole documented host of *Epichloë* to date. Host grasses infected with *Epichloë* exhibit no discernible disease symptoms and serve as habitats for the endophyte’s life cycle (Siegel et al., 1987). *Epichloë* relies on host grasses for nutrients, while in turn, it contributes to the host’s resilience against external stressors. This mutualistic relationship between *Epichloë* and host grasses underscores its ecological significance. Research on *Epichloë* species gained scholarly attention in the late 1970s, particularly following Bacon et al.’s (1977) findings on the production of alkaloids by *Epichloë*, which could induce toxic reactions in herbivorous livestock. Subsequent research deepened our understanding of the relationship between *Epichloë* and its host grasses, revealing the diverse effects of *Epichloë* on its hosts and ecological roles. Furthermore, *Epichloë* species have emerged as important agricultural microbial resources (O'Keeffe et al., 2022). *Epichloë* species employ various modes of transmission, including vertical and horizontal transmission. Vertical transmission occurs asexually through the seeds of the mother plant lineage, while horizontal transmission occurs sexually via ascospores. *Epichloë* species transmitted horizontally may exhibit antagonistic characteristics with their hosts, leading to “choke” or “cattail” disease (White, 1997). Some *Epichloë* species can be facultatively transmitted, utilizing both ascospores and seeds for transmission (Tintjer et al., 2008; Gundel et al., 2017). However, vertical transmission remains the predominant mode reported for *Epichloë* species (Chen et al., 2015).

The production of alkaloids by symbiotic *Epichloë* is a significant area of research within the field of endophytic fungi. Alkaloids play a crucial role in deterring herbivorous animals from consuming host grasses. *Epichloë* species can synthesize four types of alkaloids: peramine, ergot alkaloids, indole-diterpenes, and lolines (Bush et al., 1997). These alkaloids exhibit varying degrees of toxicity to insects and livestock, with ergot alkaloids and indole-diterpenes being toxic to both, while peramine and lolines are toxic to insects but safe for livestock (Schardl et al., 2013; Roberts and Lindow, 2014; Guerre, 2015; Philippe, 2016). Utilizing *Epichloë* for the improvement of plant germplasm has gained traction in recent years. Researchers have identified novel strains of *Epichloë* that impart robust insect resistance without causing harm to livestock. These strains have been introduced into various grasses or crops through artificial inoculation, thereby directly or indirectly assisting host grasses by producing alkaloids and influencing interspecific and intraspecific competition (Clay and Holah, 1999; Hare, 2011; Li et al., 2014). Vertical transmission is essential for maintaining genetic stability and is a prerequisite for plant breeding (Becker and Leon, 1988). However, challenges such as host specificity limit the success of artificial inoculation with *Epichloë* species (Becker et al., 2018). *Psathyrostachys*, a small genus within the family Poaceae, commonly known as the grass family, was described by Nevski (1934). It includes several species of perennial grasses native to Asia, particularly China and Mongolia. These grasses are valued for their forage qualities and ability to withstand harsh environmental conditions, making them important resources for livestock grazing and soil stabilization in arid and semi-arid regions. So far, eight recognized species of *Psathyrostachys*, with two subspecies, have been identified, six of which were previously classified under the genera *Hordeum* and *Elymus* (Baden et al., 1989; Baden, 1991). Among the species, *P. lanuginosa* has garnered attention due to its valuable biological characteristics, including early maturity, high quality, and stress resistance (Kang et al., 2011). As aforementioned, *Epichloë* species have been identified as endophytic fungi of various grass plants, particularly those belonging to the family Poaceae. However, to date, *Epichloë* species have not been detected in *P. lanuginosa*. In the present study, we isolated and identified *Epichloë* strains as endophytes of *P. lanuginosa* using morphological keys and phylogenetic analysis. Furthermore, we conducted alkaloid profiling of four strains of *Epichloë*. This research contributes to our understanding of *Epichloë* species from a new host, *P. lanuginosa*, and expands our knowledge of the host diversity of *Epichloë* species.

## 2 Materials and methods

### 2.1 Collection of plant samples and isolation of endophytic fungi

Plant samples of *P. lanuginosa* were collected from Yulin, Shaanxi Province, China (N37°32′04″, E108°52′06″; August 2021) and Lanzhou, Gansu Province, China (N36°07′10″, E103°42′05″; August 2021). Following collection, the plant samples were promptly transported to the laboratory for microscopic assessment of *Epichloë* species infection by staining the plant stalks with aniline blue (Li, 2005). Roughly 50% of the seeds obtained from the plant samples were allocated for propagation purposes, while the remaining were designated for the isolation of *Epichloë* species. To achieve endophyte isolation, the seeds were subjected to surface sterilization using 70% ethanol for 3 min, followed by treatment with a 5% sodium hypochlorite solution for an equivalent duration. The sterilized seeds were subsequently subjected to a triple wash with sterile water, and their surface moisture was removed by blotting with sterile filter paper. These sterilized seeds were then introduced into PDA media, which was supplemented with 100 μg mL^−1^ of ampicillin and 50 μg mL^−1^ of streptomycin sulfate. Finally, the PDA plates were wrapped with sealing film and incubated in darkness at a temperature of 22°C. Throughout this period, contaminated seeds were removed, and uncontaminated seeds were monitored until the emergence of endophytic fungi. Finally, four strains: PF5, PF9, QG2, and QG4 were obtained. Among them, strains PF5 and PF9 were isolated from the *Psathyrostachys* grown in Lanzhou, while strains QG2 and QG4 were isolated from the *Psathyrostachys* grown in Yulin.

### 2.2 Morphological examination

The morphological examination of endophytes was conducted on PDA plates. Using a sterile puncher, 0.4 cm diameter mycelial plugs were taken from a 30-day-old colony and placed in the center of the PDA medium. The plates were then sealed with sealing film and cultured at 22°C in the dark for 32 days. After the incubation period, colony morphology was observed, recorded, and photographed, and a comparison was made to determine if there were any differences in colony morphology between the different strains. Similarly, the growth rate of the strains was measured using 0.4 cm diameter mycelia plug taken from the 30-day-old colonies. Each strain was tested on six replicate plates, which were then placed in the center of the PDA medium and cultured under dark conditions at both 22°C and 25°C, respectively. Weekly measurements of colony diameter were conducted for 8 weeks using the ‘crossing method’. The PDA medium was also used for observing and measuring conidia and the length of conidiogenous cells. After all the strains were cultured for 2 weeks, sterile coverslips were inserted into the PDA medium at a 45° angle, and the PDA plates were sealed with sealing film. Culture continued until the mycelia of strains grew to the surface of the coverslips. The coverslips were then removed, placed on a glass slide with a drop of toluidine blue solution, and examined using an automated upright fluorescence microscope (Olympus, BX63). Measurements of 50 conidia and 30 conidiogenous cells of each isolate were taken, including their width and length.

### 2.3 Phylogenetic analysis

Purified strains were cultured on PDA medium for 2 weeks, and the mycelium was collected into 2 mL tubes by gently scraping the surface of PDA plates with a sterile glass rod. The total DNA of the endophytic strains was extracted following the manufacturer’s instructions using a fungal DNA extraction kit (Omega, Beijing, China). After extraction, the DNA was stored at -20°C until further use. Species identification of the fungal strains was conducted by direct sequencing of the housekeeping genes *tefA* and *tubB* using the extracted DNA with the highest concentration. The primer sets tef1-exon 5u-1 (GGCAGCGATAATCAGGATAG) and tef1-exon 1d-1 (GGGTAAGGACGAAAAGACTCA) were employed for *tefA* (Moon et al., 2002), while tub2-exon 4u-2 (GTTTCGTCCGAGTTCTCGAC) and tub2-exon 1d-1 (GAGAAAATGCGTGAGATTGT) were used for *tubB* (Moon et al., 2007). PCR reactions were performed in 25 μL volumes, consisting of 12.5 μL 2× SanTaq PCR Master Mix, 9.5 μL ddH_2_O, 1 μL DNA (40 ng μL^−1^), and 1 μL each of the forward and reverse primers (10 μM). The PCR protocol included an initial denaturation step at 94°C for 5 min, followed by 34 cycles of denaturation at 94°C for 30 s, annealing at 55°C (*tefA*) or 45°C (*tubB*) for 30 s, extension at 72°C for 1 min, and a final extension at 72°C for 10 min, with a hold at 4°C. Sequencing of all PCR products was conducted by Shanghai Sangon Biology Engineering Technology and Service Co., Ltd. The obtained sequences were compared against published nucleotide sequences using Blast on the NCBI website to preliminarily determine their classification within the *Epichloë* genus. Subsequently, the sequences were aligned with other *Epichloë* species using MAFFT software (v. 7.505) (Dereeper et al., 2008), and poorly aligned regions were removed with Gblocks v. 0.91b (Katoh and Standley, 2013). Substitutional saturation of the sequences was assessed using DAMBE software (Xia, 2017), maximum-likelihood phylogenetic trees (ML) with substitution model TNe + G4 were constructed using IQ-tree software (v. 2.2.0) (Minh et al., 2020), with a bootstrap value of 1,000. The *Epichloë* species names, strain names, hosts, and accession numbers used for the construction of phylogenetic trees are listed in Table 1.

**Table 1:** Accession numbers for known *tefA* and *tubB* genes used for phylogenetic analysis.

***Epichloë* species**	**Isolate**	**Host**	***tefA***	***tubB***
*E. amarillans*	906	*Agrostis perennans*	AF457506	-----
	ATCC 200744	*Agrostis hiemalis*	AF231192	-----
	E4668	*Agrostis hyemalis*	-----	KF042042
	273	*Agrostis hiemalis*	-----	AF457466
*E. aotearoae*	e899	*Echinopogon ovatus*	KP689565	-----
	829	*Echinopogon ovatus*	AF323391	-----
*E. baconii*	9707	*Agrostis tenuis*	KF811547	KF811579
	ATCC76552	*Agrostis stolonifera*	AF231193	-----
	E248	*Agrostis stolonifera*	-----	L06961
	E242	*Agrostis capillaris*	-----	L78279
*E. brachyelytri*	ATCC 201560	*Brachyelytrum erectum*	-----	AF250736
	ATCC 201561	*Brachyelytrum erectum*	-----	AF062427
*E. bromicola*	9633	*Bromus erectus*	AY033359	-----
	Rnj4301	*Elymus kamoji*	DQ134034	-----
	T36	*Elymus tangutorum*	MT905328	-----
	T29	*Elymus tangutorum*	MT905321	-----
	T23	*Elymus tangutorum*	MT905315	-----
	1511	*Elymus dahuricus*	KX219727	-----
	E7626	*Elymus dahuricus*	MF838712	-----
	XE1-3B	*Hordeum bogdanii*	MW961392	-----
	NI_201203	*Elymus excelsus*	KJ585717	-----
	NI_201201	*Elymus excelsus*	KJ585716	-----
	B3	*Hordeum bogdanii*	MW961387	-----
	AL0426/2	*Thinopyrum intermedium*	-----	KP689571
	303	*Leymus chinensis*	-----	JN819479
	229	*Leymus chinensis*	-----	JN819478
	E501	*Bromus erectus*	-----	L78289
	8918/1	*Bromus benekenii*	-----	AY033369
	Rnj4201	*Elymus kamoji*	-----	DQ134039
	362	*Hordelymus europaeus*	-----	AF457488
	Ebo201558	*Bromus ramosus*	-----	KC936102
	E502	*Bromus erectus*	-----	L78290
	0814/1	*Agropyron repens*	-----	GU325782
	NI_201216	*Elymus tangutorum*	-----	KJ585739
	NI_201302	*Elymus nutans*	-----	KJ585743
	NI_201209	*Elymus tangutorum*	-----	KJ585735
	3635	*Hordeum brevisubulatum*	-----	AY137612
	PF5	*Psathyrostachys lanuginosa*	OR727355	PP001830
	PF9	*Psathyrostachys lanuginosa*	OR750671	PP001831
	QG2	*Psathyrostachys lanuginosa*	PP001834	PP001832
	QG4	*Psathyrostachys lanuginosa*	PP001835	PP001833
*E. calamagrostidis*	AL9618/1	*Calamagrostis villosa*	MW283394	MW283357
	AL0430/1	*Calamagrostis villosa*	MW283393	MW283356
	AL0908	*Calamagrostis purpurea*	-----	MW283355
*E. clarkii*	ATCC 200742	*Holcus lanatus*	AF231206	-----
*E. elymi*	ATCC 201551	*Elymus virginicus*	KP689557	KF042052
	ATCC 201553	*Elymus virginicus*	AF457498	-----
	E56	*Elymus canadensis*	-----	L06962
*E. festucae*	ATCC90661	*Festuca rubra* subsp. *rubra*	AF231210	-----
	E2368	*Festuca rubra*	-----	KF042044
	F11	*Festuca longifolia*	-----	AY722412
	ATCC 90660	*Festuca rubra* subsp. *commutata*	AF231214	-----
*E. festucae* var. *lolii*	135	*Lolium perenne*	AF457540	-----
*E. ftanensis*	AL1614/1	*Calamagrostis arundinacea*	MW283389	MW283352
	AL2015/1	*Calamagrostis arundinacea*	MW283391	MW283354
	AL1614/2	*Calamagrostis arundinacea*	-----	MW283353
*E. glyceriae*	ATCC 200747	*Glyceria striata*	AF231216	-----
	E277	*Glyceria striata*	KP689560	KF042046
	E2772	*Glyceria striata*	-----	L78276
*E. poae*	BlaTG-1	*Bromus laevipes*	JX679188	-----
	*	*Poa secunda* subsp. *juncifolia*	JQ756452	-----
	AL0507	*Poa nemoralis*	-----	MW662267
	*	*Poa secunda* subsp. *juncifolia*	-----	JQ756453
*E. scottii*	DSM_111775	*Melica uniflora*	MZ224336	-----
	DSM_112488	*Melica uniflora*	MZ224334	-----
*E. sinca*	Rxy6106	*Elymus kamoji*	FJ189478	-----
*E. sinensis*	MHLZU-FS57	*Festuca simensis*	KX685662	-----
*E. sylvatica*	ATCC 200748	*Brachypodium sylvaticum*	AF231218	-----
	Brhs6402	*Brachypodium sylvaticum*	EU709884	-----
	E354	*Brachypodium sylvaticum*	-----	L78278
	E503	*Brachypodium sylvaticum*	-----	L78291
*E. typhina*	E348	*Phleum pratense*	AF231227	
	9636	*Poa trivialis*	-----	KF811578
	POR46	*Lolium perenne*	-----	KY997148

^*^No isolate name was found in the corresponding literature; ----, Not used in this study.

### 2.4 Alkaloid gene profiling

PCR analysis was conducted to assess the presence of 35 genes associated with the biosynthesis of four major groups of alkaloids in all endophytic strains. Among the 35 genes, one gene is involved in peramine biosynthesis, 14 genes are involved in ergot alkaloid biosynthesis, 11 genes are involved in indole-diterpene biosynthesis, and 11 genes are involved in loline alkaloid biosynthesis. Additionally, the mating-type genes of the strains were also determined using PCR. Details of the 46 pairs of primers used are provided in Supplementary Table S1. PCR amplification was conducted in 25 μL reaction volumes. The protocol included an initial pre-denaturation step at 94°C for 1 min, followed by 30 cycles of denaturation at 94°C for 15 s, annealing at 56°C for 30 s, and extension at 72°C for 1 min. A final extension step at 72°C for 10 min was performed, followed by holding at 4°C. Subsequently, PCR products were analyzed by 1.5% agarose gel electrophoresis to determine the presence of the target genes in the strains.

## 3 Results

### 3.1 Characteristics of endophytes from *Psathyrostachys lanuginosa*

**Figure 1:**
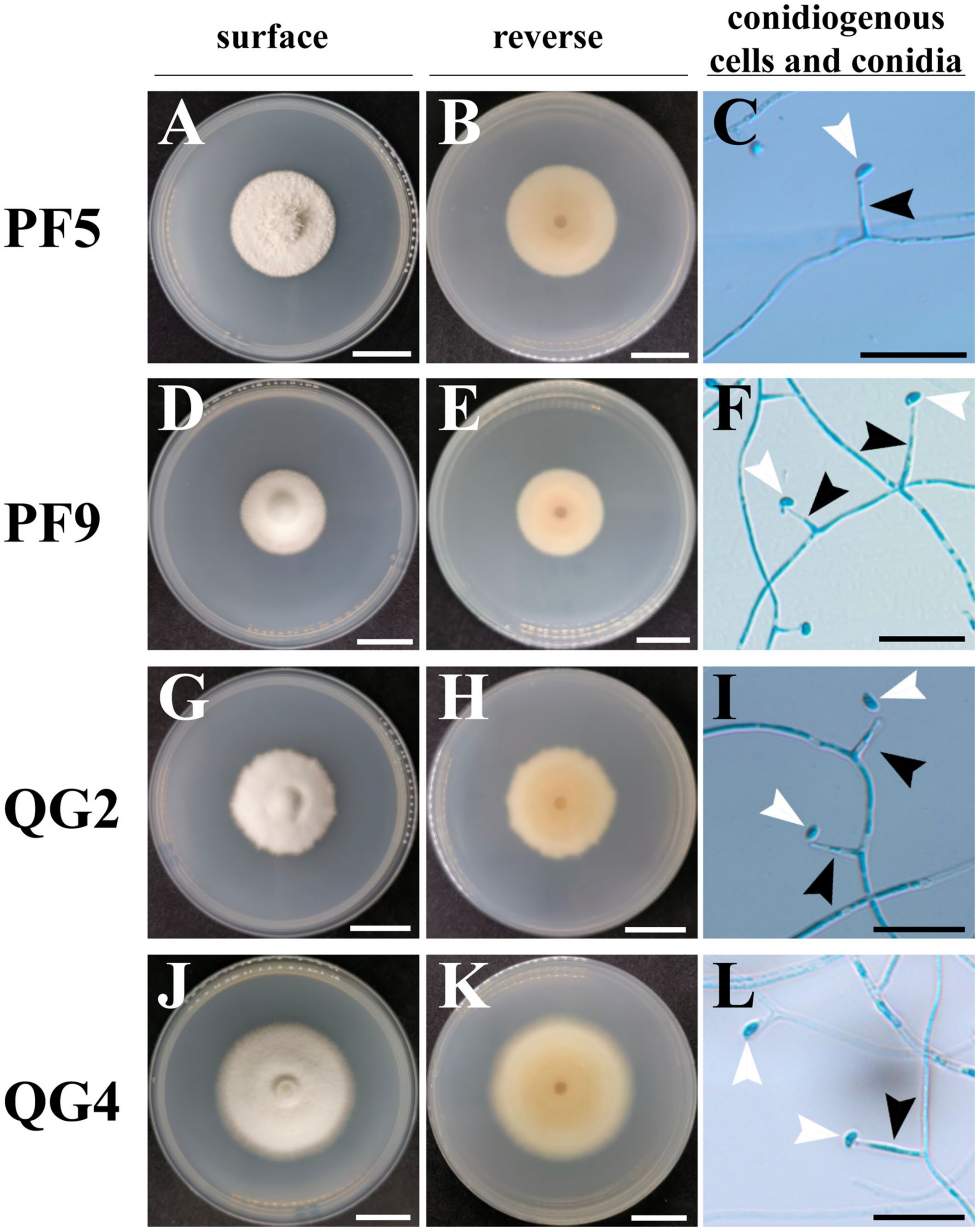
Colony morphology, conidiogenous cells, and conidia of *Epichloë* strains from *Psathyrostachys lanuginosa*. The colony is from cultures grown on PDA at 22°C for 32 days. **(A,D,G,J)** The surface view of colonies of strains PF5, PF9, QG2, and QG4; **(B,E,H,K)** The reverse view of colonies of strains PF5, PF9, QG2, and QG4; **(C,F,I,L)** The micrographs of toluidine blue-stained conidiogenous cells (black arrow) and conidia (white arrow) of strains PF5, PF9, QG2, and QG4; white scale bars = 2 cm, black scale bars = 20 μm.

A total of four endophytic fungal strains were recovered from surface-sterilized *P. lanuginosa* samples infected by *Epichloë*. Strains PF5 and PF9 were obtained from *P. lanuginosa* in Lanzhou, while strains QG2 and QG4 originated from *P. lanuginosa* in Yulin. Although all strains exhibited typical traits of *Epichloë* endophytic fungi, slight variations were observed. Overall, the colonies of the four endophytic fungi appeared white on the front with sparse outer aerial hyphae. The central region of the colonies was yellowish, gradually fading toward the edges. However, the colony edge of isolate QG2 was irregular compared to the other three strains, isolate PF5 had a slightly tougher colony texture, and isolate QG4 displayed an obvious growth circle (Figure 1). The colonies exhibited moderate growth rates on PDA at 22°C/25°C, reaching diameters of 11.29–15.97/16.96–19.89 mm (14 days), 20.65–29.40/31.26–44.98 mm (28 days), 26.45–43.31/51.75–61.78 mm (42 days), and 30.74–54.26/54.99–65.15 mm (56 days). Conidia shapes were predominantly oval and asymmetrical, with an average size of 3.5–3.9 × 1.8–1.9 μm, and an average length of the conidiogenous cell of 10.2–11.3 μm (Table 2). There were no significant differences observed in growth rate, length of conidiogenous cells, and conidia size among the four strains studied. The morphological characteristics of the other *E. bromicola* strains listed in Table 2 included a growth rate ranging from 0.88 to 1.29 mm day^−1^, length of conidiogenous cells ranging from 8 to 29 μm, length of conidia ranging from 3.7 to 5.3 μm, and width of conidia ranging from 1.8 to 3.5 μm (Table 2). When compared to previously reported *E. bromicola* endophytes, the morphological features (growth rate, length of conidiogenous cells, and conidia size) of the four strains examined in this study were slightly smaller but still fell within the normal range.

**Table 2:** Morphological characteristics of *Epichloë bromicola* endophytes.

Isolate	Host	Growth on PDA (mm/day)	Length of conidiogenous cell (μm)	Conidia Size (μm)		References
				Length	Width	
PF9	*Psathyrostachys lanuginosa*	1.23 ± 0.05a (25°C)	12.3 ± 0.57a	3.6 ± 0.07a	1.8 ± 0.04a	This study
PF5		1.33 ± 0.04a (25°C)	11.2 ± 0.53a	3.9 ± 0.06a	1.8 ± 0.03a	This study
QG2		1.02 ± 0.09ab (25°C)	10.9 ± 0.50a	3.5 ± 0.07a	1.8 ± 0.04a	This study
QG4		1.17 ± 0.02a (25°C)	10.2 ± 0.63a	3.7 ± 0.09a	1.9 ± 0.06a	This study
	*Leymus chinensis*	1.7 ± 0.07 (25°C)	29.0–31.0	5.3 ± 0.1	3.5 ± 0.1	Zhu et al. (2013)
*E. bromicola*	*Bromus erectum*	2.29–2.48 (24°C)	8–23	3.8 ± 0.4	2.0 ± 0.3	Leuchtmann and Schardl (1998)
	*Bromus ramosus*	0.90 (24°C)	nt	4.2 ± 0.5	2.0 ± 0.3	
	*Bromus ramosus*	nt	nt	3.7–4.8	1.8–2.3	Brem and Leuchtmann (2003)
	*Hordeum bogdanii*	1.21 ± 0.1	14.0 ± 3.5	4.6 ± 0.4	2.7 ± 0.3	Yi et al. (2018)
	*Hordeum bogdanii*	0.99 ± 0.1	19.5 ± 5.7	5.0 ± 0.5	2.7 ± 0.3	
	*Hordeum brevisubulatum*	0.88 ± 0.01 (25°C)	19.50 ± 1.06	5.17 ± 0.06	2.87 ± 0.17	Chen et al. (2019)
	*Hordelymus europaeus*	1.43–1.67 (24°C)	20.2 ± 4.7	4.2 ± 0.4	2.1 ± 0.2	Leuchtmann and Oberhofer (2013)

The same lowercase letter indicates no significant difference (*p* > 0.05), and different lowercase letters indicate significant differences (*p* < 0.05); nt, not tested.

### 3.2 Phylogenetic relationships

**Figure 2:**
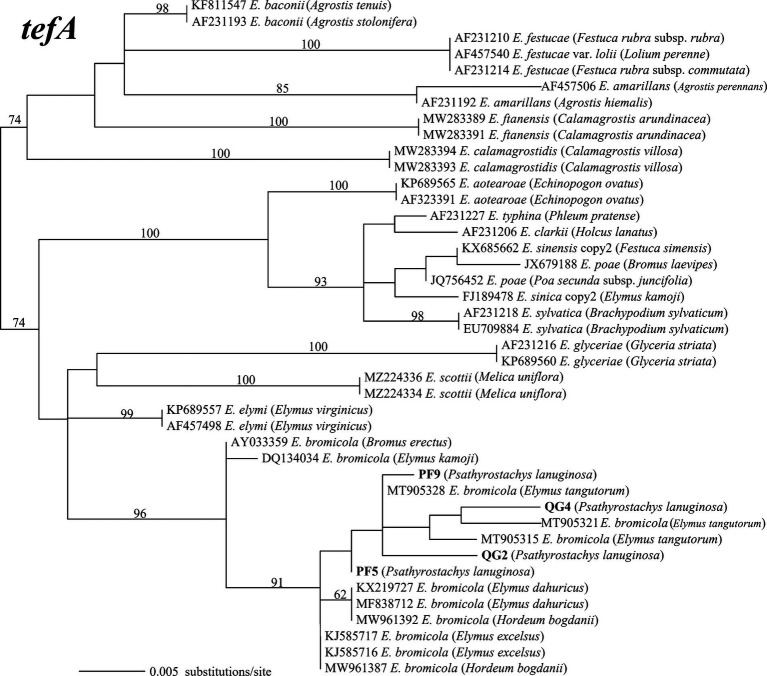
The molecular phylogeny derived from maximum likelihood (substitution model TNe + G4) analysis of the *tefA* gene from representative *Epichloë* species from *Psathyrostachys lanuginosa*. Numbers above the branches are bootstrap support percentages assessed with 1,000 replications.

**Figure 3:**
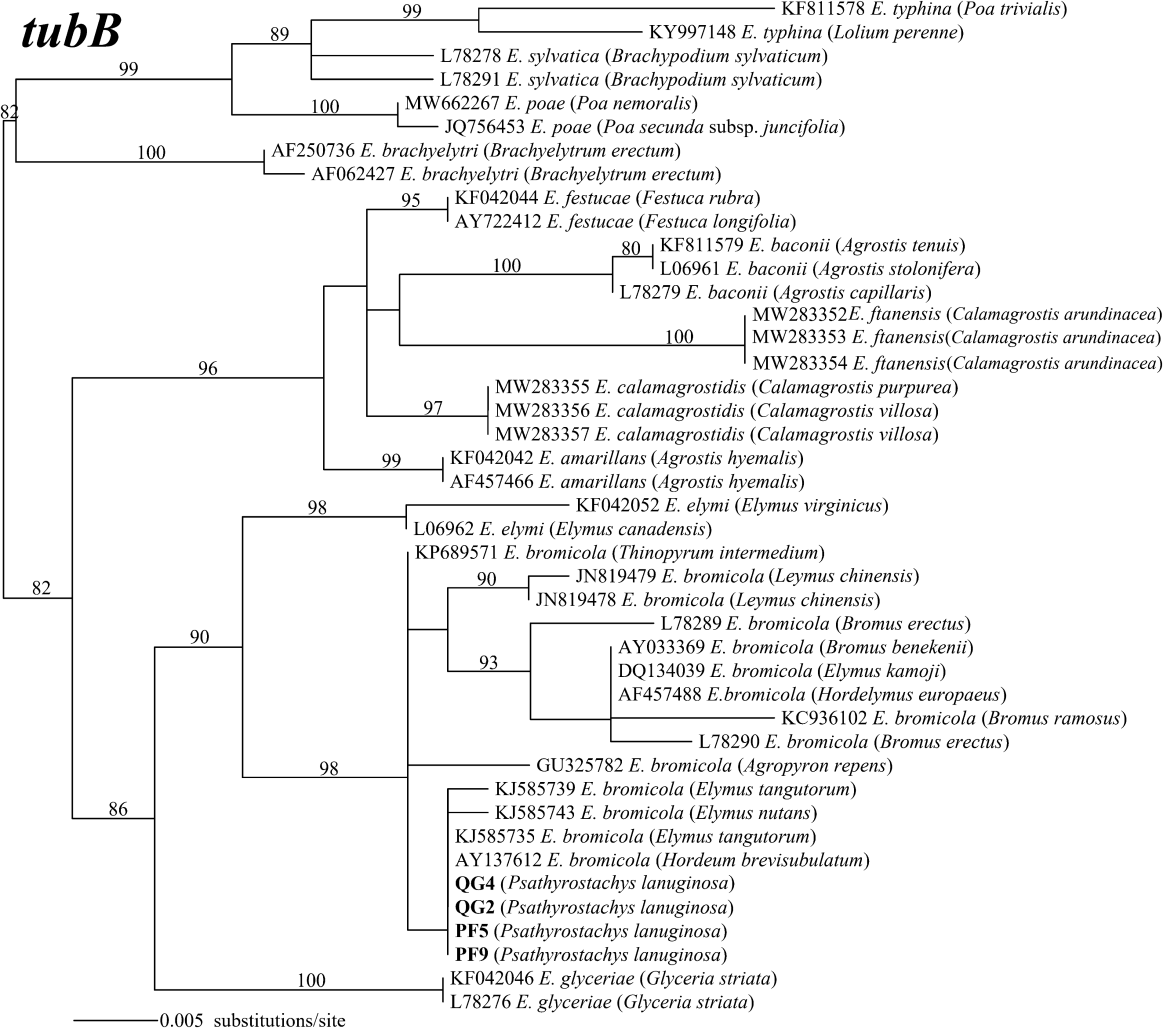
The molecular phylogeny derived from maximum likelihood (substitution model K2P + G4) analysis of the *tubB* gene from representative *Epichloë* species from the *Psathyrostachys lanuginosa*. Numbers above the branches are bootstrap support percentages assessed with 1,000 replications.

The amplified PCR products yielded single peaks in the sequencing results, indicating that the four strains belong to non-hybrid species. This classification was further reinforced by the construction of maximum likelihood phylogenetic trees using *tefA* and *tubB* gene sequences. Specifically, strains PF5 and PF9 exhibited taxonomic congruence with strains QG2 and QG4. Phylogenetic analysis, employing 42 *tefA* gene sequences, revealed that all four strains formed a distinct clade with *E. bromicola*, supported by a bootstrap value of 96% (Figure 2). Similarly, in the phylogenetic analysis based on 43 *tubB* gene sequences, all strains grouped together with *E. bromicola*, with a bootstrap value of 98% (Figure 3). Thus, our investigation confirms the identification of the endophytic strains infecting *P. lanuginosa* as *E. bromicola*.

### 3.3 Alkaloid gene profiling

All four strains exhibited differences in alkaloid synthesis genes and mating-type genes, reflecting variations in alkaloid production among the four strains as shown in Table 3. While the synthesis genes for ergot and loline alkaloids were consistent across all strains, disparities were observed in the synthesis genes of peramine and indole-diterpene alkaloids (Table 3). Notably, all four strains exclusively contained the *lolC* gene within the genes responsible for loline alkaloid synthesis, suggesting a potential deficiency in synthesizing loline alkaloids. Among the 14 genes implicated in ergot alkaloid synthesis, all four strains possessed genes *dmaW*, *easF*, *easE*, *easC*, *easD*, *easA*, *easG*, *cloA*, *lpsA*, *lpsB*, and *easH*, while lacking genes *lpsC*, *easO*, and *easP*, which encode enzymes for a separate branch to ergonovine, lysergic acid alpha-hydroxylamide, and to ergine. Consequently, the four strains demonstrated potential for synthesizing chanolavine I (CC), D-lysergic acid, and ergovaline (ERV), albeit lacking the potential for synthesizing ergonovine (EN) or lysergic acid α-hydroxyethylamide (LAH). Within the eight domain structures of the peramine synthetase-encoding gene, *ppzA* (formly *perA*), all four strains harbored six domains, including *ppzA*–A1, *ppzA*–T1, *ppzA*–C, *ppzA*–A2, *ppzA*–M, and *ppzA*–T2. However, strains PF5 and PF9 possessed the *ppzA*–R domain (representing allele *ppzA*-1), while strains QG2 and QG4 harbored the *ppzA*–ΔR domain (representing allele *ppzA*-2) in reverse. *ppzA*-∆R means *ppzA* from which the R-domain was deleted, the implication of the deletion is that the final enzymatic step from the diketopiperazine to peramine is missing in the ∆R versions, such that pyrrolopyrazine-1,4-diones are produced instead of peramine. Recent studies have indicated its capacity to encode different metabolites and confer protective effects on the host (Berry et al., 2019). Regarding the 11 genes within the *IDT/LTM* clusters, strains PF5 and PF9 contained nine of them, including *idtG, idtB, idtM, idtC, idtS, idtP, idtO, idtF,* and *idtK,* suggesting their potential to synthesize paspaline, terpendole I, paxilline (PAX), and terpendole K (TDK), but not lolitrem B (LTM) theoretically. Conversely, strains QG2 and QG4 only possessed three genes (*idtM*, *idtS*, and *idtK*) related to indole-diterpene synthesis, probably rendering them incapable of synthesizing any type of indole-diterpene alkaloids due to the absence of the pivotal gene *idtG* in the *IDT/LTM* cluster.

**Table 3:** Profiling of alkaloid genes to determine alkaloid chemotypes.

Gene		PF5	PF9	QG2	QG4
Segments of *ppzA* Gene	*ppzA*–A1	+	+	+	+
	*ppzA*–T1	+	+	+	+
	*ppzA*–C	+	+	+	+
	*ppzA*–A2	+	+	+	+
	*ppzA*–M	+	+	+	+
	*ppzA*–T2	+	+	+	+
	*ppzA*–R	−	−	+	+
	*ppzA*–ΔR	+	+	−	−
Loline (LOL) Genes	*lolC*	+	+	+	+
	*lolF*	−	−	−	−
	*lolD*	−	−	−	−
	*lolT*	−	−	−	−
	*lolA*	−	−	−	−
	*lolU*	−	−	−	−
	*lolO*	−	−	−	−
	*lolE*	−	−	−	−
	*lolN*	−	−	−	−
	*lolM*	−	−	−	−
	*lolP*	−	−	−	−
Ergot Alkaloid (EAS) Genes	*dmaW*	+	+	+	+

*easF*	+	+	+	+
	*easE*	+	+	+	+
	*easC*	+	+	+	+
	*easD*	+	+	+	+
	*easA*	+	+	+	+
	*easG*	+	+	+	+
	*cloA*	+	+	+	+
	*lpsA*	+	+	+	+
	*lpsB*	+	+	+	+
	*easH*	+	+	+	+
	*lpsC*	−	−	−	−
	*easO*	−	−	−	−
	*easP*	−	−	−	−
Indole–Diterpene (IDT/LTM) Genes	*idtG*	+	+	−	−

*idtB*	+	+	−	−
	*idtM*	+	+	+	+
	*idtC*	+	+	−	−
	*idtS*	+	+	+	+
	*idtP*	+	+	−	−
	*idtQ*	+	+	−	−
	*idtF*	+	+	−	−
	*idtK*	+	+	+	+
	*idtE*	−	−	−	−
	*idtJ*	−	−	−	−
Mating–Type Genes	*mtAC*	+	+	−	−
	*mtBA*	−	−	+	+

## 4 Discussion

In this study, we identified *P. lanuginosa* as a previously unreported host of *Epichloë* species, Four endophytic fungal strains of *Epichloë* were isolated from *P. lanuginose* from two distinct geographical locations, i.e., Yulin, Shaanxi Province and Lanzhou, Gansu Province, China. Morphological and phylogenetic analyses based on *tefA* and *tubB* sequences confirmed the taxonomic status of these four strains as *E. bromicola*. Furthermore, we elucidated the presence of alkaloid synthesis genes within these four *E. bromicola* strains. *Epichloë bromicola* exhibits a broad host range within the Poaceae family. Previous studies have identified this endophytic fungus in various grass species, including *Hordeum* (Iannone et al., 2015; Yi et al., 2018; Chen et al., 2019), *Leymus* (Zhu et al., 2013), *Elymus* (Li et al., 2006; Song and Nan, 2015; Shi et al., 2017), *Bromus* (Leuchtmann and Schardl, 1998; Groppe et al., 1999), and *Agropyron* (Lembicz et al., 2010). In a study by Shi et al. (2017), *E. bromicola* isolated from *E. dahuricus* revealed that all but one isolate out of 10 belonged to mating type A (MTA). Phylogenetic analysis of seven strains using *tefA* and *tubB* showed that six grouped together, while the seventh, the only mating type B (MTB) strain, grouped with those from *E. kamoji*, known to be sexual (Li et al., 2006). Li et al. (2006) conducted a study where they isolated eight strains of *E. bromicola* from *E. kamoji* native to China. Among these strains, two were classified as MTA, while the remaining six were categorized as MTB. Similarly, Yi et al. (2018) analyzed *E. bromicola* from six different seed accessions, all of which were MTA. Furthermore, Chen et al. (2019) found that three *E. bromicola* isolates that were symbiotic with *H. brevisubulatum*, and all were MTA. These studies collectively suggest that mating type diversity is extremely low in *E. dahuricus*, *H. bogdanii*, and *H. brevisubulata*. In the current research, we found two MTA and two MTB isolates from *P. lanuginosa*. This discovery suggests the presence of a sexual population in *P. lanuginosa*. However, stromata were not observed on this host under natural conditions. This novel endophyte-grass combination raises questions about the widespread occurrence of this association and warrants further investigation. In some species with sexual *Epichloë*, stromata rarely form, and even if sexual reproduction is infrequent, it may still play a significant role in endophyte diversification.

Significant variation in alkaloid biosynthetic potential among *E. bromicola* isolates from different or the same hosts has been observed in previous studies (Shi et al., 2017; Chen et al., 2019). This phenomenon was further supported in our study. We investigated the alkaloid biosynthesis gene profiles of four *E. bromicola* strains (PF5, PF9, QG2, QG4). All strains lacked genes necessary for loline alkaloid synthesis but possessed the potential to produce ergot and peramine alkaloids. Genetic polymorphisms within the *ppzA* gene results in differential peramine vs. pyrrolopyrazine-1,4-diones production among the strains (Berry et al., 2019). Furthermore, PF5 and PF9 harbored genes potentially involved in indole-diterpene alkaloid biosynthesis, absent in QG2 and QG4 strains. The QG2 and QG4 strains lacked *idtG* required for paspaline production, suggesting limitations in synthesizing any type of indole-diterpene alkaloids. Similar observations were reported for *E. bromicola* isolated from *Elymus dahuricus*, highlighting remarkable intraspecific diversity within *E. bromicola* regarding its alkaloid biosynthetic potential (Shi et al., 2017). This diversity appears to be influenced by both host plant species and genetic polymorphisms within the fungal population.

The genus *Psathyrostachys*, a perennial member of the Triticeae tribe, has primarily been investigated in the context of agricultural applications. Unlike common wheat (*Triticum aestivum*) with its A, B, and D genomes, or other Triticeae members with I, H, R, St, P, E, and W genomes, the entire *Psathyrostachys* genus possesses a distinct Ns genome (Hsiao et al., 1986). This unique genetic makeup offers a valuable resource for the improvement of common wheat due to the presence of beneficial traits and genes (Cao et al., 2008; Ma et al., 2016). For example, *P. huashanica*, is an endemic species found in China’s Qinling Mountains, exemplifies the potential of this genus. This species exhibits cold, drought, and barren tolerance, early maturity, high grain quality, and resistance to stripe rust, take-all, and scab (Shu et al., 1991; Du et al., 2014; Ma et al., 2016). These characteristics position it as a significant source of novel genetic diversity within Triticeae. Furthermore, distant hybridization techniques have enabled the successful transfer of superior high-molecular-weight (HMW) gliadin genes from the *Psathyrostachys* Ns genome into common wheat (Zhao et al., 2010). These findings underline the agricultural importance of *Psathyrostachys*, independent of its potential as a microbial resource. The present study unveils a novel symbiotic association between *P. lanuginosa* and *E. bromicola*, a finding with significant implications. While *Epichloë* symbioses typically confer benefits to host grasses, no prior reports documented such interactions within *Psathyrostachys* (Song et al., 2015). The combination of *Psathyrostachys*, known for its exceptional traits, with an symbiotic *Epichloë* raises the possibility of further enhanced performance, considering the well-documented benefits provided by *Epichloë* symbioses in other grasses (Song et al., 2015). However, the absence of previous research on *Psathyrostachys*-*Epichloë* interactions and the exclusion of growth and stress resistance evaluations in this study necessitate further investigation.

The study of *Epichloë* species in grasses has emerged as a significant discipline in research history. Our understanding of *Epichloë* species has evolved considerably over time, transitioning from early incidences of livestock poisoning to contemporary insights gained from diverse perspectives (Bacon et al., 1977). As our comprehension of *Epichloë* species continues to advance, it also presents an increasing array of challenges. The utilization of *Epichloë* species in plant breeding has been progressively adopted due to their host stress resistance traits and the detectability and facile screening of alkaloids synthesized by these endophytes. One strategy involves the inoculation of *Epichloë* species that do not synthesize harmful alkaloids into other grass species, thereby generating novel germplasm with desirable attributes (White et al., 2019). However, practical outcomes are often suboptimal. Firstly, the success rate of *Epichloë* species inoculation is limited by their host specificity. This is influenced by factors such as inoculation technique, plant genotype, and *Epichloë* strains, necessitating ongoing optimization and adjustment of inoculation methods (Becker et al., 2018). Secondly, some materials successfully inoculated with *Epichloë* species may exhibit poor performance, such as severe stunting (Simpson et al., 2014). Nonetheless, it’s noteworthy that while some materials artificially inoculated with *Epichloë* have demonstrated successful performance, there exists a potential barrier to transmission across generations due to incompatibility between certain *Epichloë* species and specific grasses. Although the underlying mechanisms are not fully understood, this underscores the importance of exploring the compatibility of *Epichloë* species with different grasses in future endeavors. Importantly, numerous successful cases demonstrate the utility of *Epichloë* species in plant germplasm innovation. For instance, researchers have utilized renowned *Epichloë* strains AR1, AR37, and NEA2 to cultivate numerous commercially viable grass cultivars, accounting for more than 70% of proprietary seed sales in New Zealand a decade ago (Caradus et al., 2013). Experimental manipulation of *Epichloë* species through fungal culture and inoculation suggests that the degree of genetic similarity between native and novel host plants positively correlates with the likelihood of establishing a mutually beneficial symbiotic relationship (Simpson and Mace, 2012). To date, no reports have been found of *Epichloë* species infecting cereal crops naturally. Given the significance of cereal crops, there is a growing interest in exploring *Epichloë* species as a means to expedite the cultivation of novel cereal crop varieties with exceptional traits (Kang et al., 2011). Several researchers have investigated the artificial inoculation of *E. bromicola* strains in cultivated barley, reporting notable successes such as enhancements in aboveground biomass, seed yield per plant, and growth period advancements. The *E. bromicola* strain utilized in these studies was isolated from wild barley and exhibited close genetic relatedness to cultivated barley (Li et al., 2021). Therefore, it is imperative to explore *Epichloë* species in wild-related species of cultivated plants. The *Epichloë* strains analyzed in this research were isolated from *Psathyrostachys*, a taxonomically related species to wheat. However, further experimentation is required to evaluate their alkaloid-producing capabilities in plants for potential application in artificial inoculation studies, which will be the primary focus of our forthcoming research.

## 5 Conclusion and future perspectives

In conclusion, we have identified *P. lanuginosa* as a previously unreported host of four endophytic fungal strains of *Epichloë* from two distinct geographical locations in China. Morphological and phylogenetic analyses confirmed the taxonomic status of these strains as *E. bromicola*, elucidating the presence of alkaloid synthesis genes within them. *Epichloë bromicola* exhibits a broad host range within the Poaceae family, with significant variation observed in alkaloid biosynthetic potential across different host species. Our investigation into the alkaloid biosynthesis gene profiles of four *E. bromicola* strains revealed variations in the presence of genes associated with alkaloid synthesis, suggesting intraspecific diversity influenced by both host plant species and genetic polymorphisms within the fungal population. Additionally, we underscore the agricultural significance of *Psathyrostachys* genus, highlighting its potential for genetic improvement of common wheat and its exceptional traits. The revelation of a novel symbiotic association between *P. lanuginosa* and *E. bromicola* prompts further exploration into the potential benefits of this interaction, emphasizing the need for future research to elucidate its implications for host grass performance. While challenges remain in optimizing *Epichloë* species inoculation and understanding the mechanisms underlying host compatibility, successful applications in plant breeding underscore the utility of these endophytes in generating novel germplasm with desirable attributes. As we continue to explore the diversity and applications of *Epichloë* species, further investigations into their interactions with different grass species and their potential for enhancing cereal crop varieties are warranted, with a particular focus on evaluating the alkaloid-producing capabilities of *Epichloë* strains isolated from wild-related species of cultivated plants. This comprehensive approach will advance our understanding of *Epichloë* biology and its agricultural applications, paving the way for the development of improved crop varieties with enhanced resilience and productivity.

## Data availability statement

The datasets presented in this study can be found in online repositories. The names of the repository/repositories and accession number(s) can be found in the article/Supplementary material.

## Author contributions

TC: Funding acquisition, Investigation, Methodology, Writing – original draft, Writing – review & editing. TW: Methodology, Software, Writing – review & editing. MD: Investigation, Writing – review & editing. KM: Formal analysis, Investigation, Writing – review & editing. CL: Funding acquisition, Project administration, Resources, Supervision, Writing – review & editing. GB: Funding acquisition, Resources, Supervision, Writing – review & editing.
