# Supplementary material for: Discovery of Epichloë as novel endophytes of Psathyrostachys lanuginosa in China and their alkaloid profiling
Source: Front Microbiol. 2024 May 23;15:1383923. doi: 10.3389/fmicb.2024.1383923 (PMC11153765; doi:10.3389/fmicb.2024.1383923)
Supplement: Supplementary file 1 [file Table_1.docx]

**Table S1** Primer sequences

| **Gene** | **Forward primer (5–3′)** | **Reverse primer (5–3′)** | **Size (bp)** |
| --- | --- | --- | --- |
| *mtAC1* | CAATGGTGGTCACCTGAGAAG | CGGTCTCATTCTTCCAGAGAGAGG | 785 |
| *mtBA1* | TCTACCGCAAGGAACGACACAATACCG | GCTTTTCCAGCAAGGCTTGCTTGACTC | 213 |
| *ppzA*–A1^2^ | ATGACGAGCTCGGAGCGAGTTG | AGACTTCCATCTGCACAGTATC | 1691 |
| *ppzA*–T1^2^ | TCGGAAAGGTCGGCTGTAC | TTGCTTCATCCCAGTCAGC | 1073 |
| *ppzA*–C^2^ | ATCCAAGACGCATATCCC | ATCATCTCGGCGGCTTCC | 878 |
| *ppzA*–A2^2^ | ACAGCTTTGCCACTCCAAG | ATCCACGCCTATGTAGCTC | 2363 |
| *ppzA*–M^2^ | GCTTGCTGCGTTTGTCAC | TGGGAAATCGGAACAAGG | 1298 |
| *ppzA*–T2^2^ | TCTTCAGGCATCGCAGGAAC | TCGGCCACCTCCAGCCTGATG | 600 |
| *ppzA*–R^2^ | AGGAAGGCATCAGGCTGG | CTAGCCTCCAGATCTTGTG | 1376 |
| *ppzA*–ΔR^2^ | TCTTCAGGCATCGCAGGAAC | GTACGGATAACCTCAAC | 742 |
| *lolC*^1^ | GTTGCCCACGGTGCGCGTCTTC | GGTCTAGTATTACGTTGCCAGGG | 462 |
| *lolF*^1^ | CTCTGATATGAAGACTCCTGAGC | GCCAAGCGGAGTTCAGATCATCC | 635 |
| *lolD*^1^ | CTCGACGTTTCAACAGATTGCAG | GTCTTTGAAGACAAGCCAGTCC | 430 |
| *lolT*^1^ | CACTGACCTCCAAGTATACTTGC | CGTCATCCCGACCTCTTTCGGAT | 551 |
| *lolA*^1^ | GAGACACTAGAGAAATGGCAGCTGC | GGCATCCATGGTGGCGAAGATGTG | 270 |
| *lolU*^1^ | CGATGGTTGGATCAGTCGTTGC | GAGCTGATGCGGCATTGGCATC | 661 |
| *lolO*^1^ | GTGAACTGGCAGTAGTCCGTATG | AATCCATGCCAGTGTCGGGAATG | 595-659 |
| *lolE*^1^ | ACCAAGCCAACGGATATCTTCGC | ACGTCTTTGGTCCGTCTTGTTAG | 587 |
| *lolN*^3^ | CCCTCCCATTCCAGATACAC | GGTTCCTTGGCTGCTTCA | 1379 |
| *lolM*^3^ | GTGAACAGCAGCGTGAAGCA | AGGGCGGTGGTCTCGTAGTC | 656 |
| *lolP*^1^ | GTTCTAAACATCGTGACTGGGC | GTTCTAAACATCGTGACTGGGC | 566 |
| *dmaW*^1^ | GTGTACTTTACTGTGTTCGGCATG | GTGGAGATACACACTTAAATATGGC | 281 |
| *easF*^1^ | GTTCTCGACATTCGTCTTGCTAC | TCAAGCTGCGAGGCACTTACGTC | 415 |
| *easE*^1^ | CAGACTCGCTTCAGATTCACAC | GTTCTGGTGTTCGTTGGCAATG | 388 |
| *easC*^3^ | CTGGAGCATATGGAGAGTTTG | AATGTTCAGGCAAACCCAGTC | 278 |
| *easD*^3^ | CTGAAGAAGTCTGTCCACGAA | TGACGATACTGCGGTTGC | 310 |
| *easA*^1^ | GCGGTTGCATTGAGAATCGCTC | ATCTACCACAAGCTTGGCGGAC | 350 |
| *easG*^1^ | AAGAAAGGTGGACCTGCCATGG | CTTCTCATCCGTTAATGTGCGG | 317 |
| *cloA*^1^ | GGATGAACTTGTCAGGTGACGAG | GTGATCAGGGATACCTTTGATTAC | 383 |
| *lpsA*^1^ | CGCTGCTCTCTGTCATGCAGAAG | GTTCCTCATCCAACATCTCGATC | 476 |
| *lpsB*^1^ | CCGTCTTCCCGTATACCGAA | TACCCACTGCCTCGAACTTG | 597 |
| *easH*^1^ | AGATATGGCATCGTGACCAGCC | GGCATGTAGCATCAAATGGTGTC | 333 |
| *lpsC*^3^ | ACGAGGGACCGAAACTAT | AATGACGAGGCTGAAAGG | 1514 |
| *easO*^3^ | GGGATGAGGCAACAAGCA | GATTCACCCGCCGACAAC | 570 |
| *easP*^3^ | CCTTGTTCTACTCTACGGTTCA | AGGCTCTTCAGGTGCTTAC | 363 |
| *idtG*^1^ | GCACAAACAATAAATTCGGCCAA | AATTTGCCCTCTGTTAAATCCTC | 383 |
| *idtB*^1^ | AACATCGCCTGGGAGCTCGTATA | CGCAGGTCCTATTTCCATCGC | 240 |
| *idtM*^1^ | GTGATCGGTGCTGACGGGGTCCA | TATCGCCATATTTGCTCCTTGCCC | 669 |
| *idtC*^1^ | GAAACTGCCAATCGAGCATA | TTCTTGCAATCATTTTGCAATTG | 403 |
| *idtS*^3^ | ATGGAAGTCACGGGAAGG | AAAGACGGGAAAGGCAGT | 374 |
| *idtP*^1^ | ATGGCTGTCATTCATACAACAGCTATG | AGCGTCCCGGACAGGCATATCTCCCA | 508 |
| *idtQ*^1^ | CTACCAGGACAGGCGTGACGTCC | CAGAGGTTTAACCCTCTTGACGC | 334 |
| *idtF*^1^ | GAATTATGTTACTCTTGGGG | AAGTTGGCACATAGGTCTTC | 227 |
| *idtK*^1^ | ATATTGAATTGCTGCGTGAGGAG | AGAGGCCAAGAAGCGGCCTGGACA | 568 |
| *idtE*^1^ | CCGAGTTTGATGACCTGCTG | TTCCGCTTCCGAGTAGACTC | 687 |
| *idtJ*^1^ | CCAAGCATCGATTTGTCACC | AATCTGATCGCCATCTTTGC | 242 |

^1^ Charlton ND, Craven KD, Mittal S, Hopkins AA, Young CA. 2012. *Epichloë canadensis*, a new interspecific *Epichloë* hybrid symbiotic with Canada wildrye (*Elymus canadensis*). Mycologia. 104(5): 1187-1199. doi: 10.3852/11-403.

^2^ Berry D, Takach JE, Schardl CL, Charlton ND, Scott B, Young CA. 2015. Disparate independent genetic events disrupt the secondary metabolism gene *perA* in certain symbiotic *Epichloë* species. Applied and Environmental Microbiology. 81(8): 2797-2807. doi: 10.1128/aem.03721-14.

^3^ Chen L. 2015. Molecular detection, genotypes and chemotypes of *Epichloë* endophytes in *Achnatherum inebrians*. Lanzhou: Lanzhou University.
